# Long-term effects of denosumab: a 10-year single-center retrospective study

**DOI:** 10.1007/s11657-026-01757-y

**Published:** 2026-08-19

**Authors:** Federico Aldegheri, Matteo Appoloni, Nicolò Passoni, Vittorio Zibordi, Maurizio Rossini, Davide Gatti, Giovanni Adami, Francesco Pollastri, Angelo Fassio, Ombretta Viapiana

**Affiliations:** Rheumatology Unit, Department of Medicine, University of Verona, Pz Scuro 10, 37134 Verona, Italy

**Keywords:** Denosumab, Bisphosphonates, Osteoporosis, Long-term therapy, Bone mineral density, Fracture risk, Real-world evidence

## Abstract

***Summary*:**

Osteoporosis weakens bones and increases the risk of fractures. Denosumab is a medication that reduces bone loss and increases bone mineral density (BMD). In this real-world study, we followed patients who received denosumab every 6 months for 10 years. We found that bone density continued to improve throughout treatment. People who had taken bisphosphonates before starting denosumab responded just as well in the long term. Blood tests showed stable reduction of bone turnover markers.

**Background:**

Denosumab produces durable BMD gains and low fracture rates in clinical trials, but long-term real-world evidence is limited, especially on the effect of prior bisphosphonate exposure and characteristics of patients who fracture during therapy.

**Methods:**

We conducted a 10-year single-center real-world retrospective study including patients receiving continuous denosumab 60 mg every 6 months. Clinical, biochemical, and densitometric data were extracted from an institutional electronic database. Longitudinal changes in BMD at total hip, lumbar spine, and femoral neck were analyzed using linear mixed-effects models adjusted for baseline BMD, age, baseline severe vertebral fractures, and glucocorticoid exposure. Bone turnover markers (CTX, ALP, PTH) were analyzed with mixed-effects models adjusted for renal function.

**Results:**

A total of 145 patients were included (mean age 70.9 years; 95.9% women). Over 10 years, BMD increased significantly at all skeletal sites: +12.9% at the total hip, + 16.3% at the lumbar spine, and +6.0% at the femoral neck. CTX and ALP showed marked and sustained reduction, whereas PTH remained stable over time. Prior bisphosphonate exposure did not modify long-term BMD trajectories at any site, and group differences in CTX converged after treatment initiation. Patients who sustained fractures showed significantly attenuated lumbar spine BMD gains, while hip sites displayed similar trajectories to non-fractured patients.

**Conclusions:**

In a real-world setting, continuous denosumab therapy for 10 years produced robust and sustained BMD gains, accompanied by a durable and stable reduction in bone turnover. Prior bisphosphonate exposure influenced only early biochemical differences and did not affect decade-long BMD responses.

## Introduction

Osteoporosis is a highly prevalent chronic condition characterized by reduced bone strength and an increased risk of fragility fractures [1, 2]. Over the past decade, denosumab has become a cornerstone therapy due to its potent and reversible inhibition of RANKL, resulting in marked reduction of bone resorption, reduction of major osteoporotic fracture, and clinically meaningful increases in bone mineral density (BMD) [3–6]. Evidence from the FREEDOM trial and its long-term extension demonstrated sustained BMD gains and low fracture rates for up to 10 years of continuous therapy, positioning denosumab as one of the most effective anti-osteoporotic agents available [7–9].

However, most of the available evidence derives from controlled trial populations, which differ substantially from the older, more heterogeneous patients encountered in routine practice [10]. Real-world data extending beyond 5–7 years remain limited, particularly regarding the long-term evolution of bone turnover markers, the clinical relevance of prior bisphosphonate exposure over a full decade of treatment, and the characteristics of patients who sustain fractures while receiving denosumab. Furthermore, whether densitometric trajectories in real-world settings replicate those observed in tightly monitored trials remains uncertain.

To address these gaps, we conducted a 10-year single-center real-world study focusing on patients who maintained continuous denosumab therapy for a full decade. Rather than estimating treatment effectiveness in the overall treated population, the study was designed to characterize the long-term densitometric and biochemical trajectories of patients who remain on sustained therapy in routine clinical practice. Specifically, we aimed to evaluate long-term changes in BMD and bone turnover markers, assess whether prior bisphosphonate exposure influences decade-long treatment trajectories, and explore whether patients who sustain fractures during therapy display distinct densitometric or biochemical patterns. By providing real-world data over a full decade of treatment, this study seeks to complement clinical trial evidence and inform long-term management strategies.

## Methods

### Study design and setting

This was a retrospective, real-world observational study conducted at the Metabolic Bone Disease Clinic of the Integrated University Hospital of Verona. The study aimed to assess the long-term effectiveness and safety of continued denosumab therapy.

### Participants and data collection

All patients managed at the clinic with a clinical indication for denosumab therapy (60 mg every 6 months) for at least 10 years were included. Clinical, biochemical, and densitometric data were retrieved from the institutional electronic database, which systematically records longitudinal information on anti-osteoporotic treatments and serial densitometric assessments.

### Eligibility criteria

Adults with osteoporosis who had received denosumab 60 mg every 6 months for a continuous period of at least 10 years, and who had a baseline dual-energy X-ray absorptiometry (DXA) scan together with at least one follow-up densitometric assessment within the tenth year of therapy, were considered eligible.

Exclusion criteria included advanced renal impairment (eGFR < 30 mL/min/1.73 m^2^) or dialysis, metabolic bone diseases other than osteoporosis (such as osteomalacia or uncontrolled primary hyperparathyroidism), previous or concomitant use of anabolic agents during the 10-year observation period, active primary or secondary bone malignancies, inappropriate discontinuation of denosumab beyond the recommended dosing interval, and insufficient densitometric data to reconstruct the full 10-year trajectory.

### Procedures

At baseline, clinical and biochemical data were collected, including age, body mass index (BMI), comorbidities, smoking status, detailed history of fragility fractures (site and number), chronic glucocorticoid use (at least ≥ 3 months of glucocorticoid), and key markers of calcium-phosphate metabolism and renal function. Prior bisphosphonate exposure was recorded; no formal washout period was applied, and patients were generally switched directly to denosumab in routine clinical practice.

Patients received denosumab every 6 months for at least 10 consecutive years. DXA was performed at baseline and every 2 years, assessing the lumbar spine, femoral neck, and total hip. BMD (g/cm^2^) and *T*-score were measured. All DXA scans were performed using a Hologic 4500 densitometer (Hologic Inc., USA), following manufacturer recommendations and the International Society for Clinical Densitometry (ISCD) guidelines [11]. The coefficient of variation was 1.0% for lumbar spine and 1.2% for the total hip. Least significant change (LSC) values for lumbar spine and femoral BMD were derived using the formula LSC = 2.77 × coefficient of variation (corresponding to 2.77% for the lumbar spine and 3.32% for the total hip).

Fasting serum samples were collected in the morning, according to standard clinical practice, for the assessment of C-terminal telopeptide of type I collagen (CTX), total alkaline phosphatase (ALP), parathyroid hormone (PTH), and 25-hydroxyvitamin D (25OHD). All laboratory analyses were performed at the central laboratory of the Verona University Hospital, which participates in internal and external quality-control programs to ensure analytical reliability.

### Endpoints

#### Primary endpoint

The primary endpoint was the percent change in total hip BMD after 10 years of continuous denosumab therapy compared with baseline. Total hip BMD was selected as a validated surrogate endpoint because it provides a robust and clinically meaningful indicator of long-term therapeutic response, is less prone to artefactual variability than lumbar spine measurements, and shows a stronger correlation with fracture risk [12].

#### Secondary endpoints

Secondary endpoints included percent change in BMD at the lumbar spine and femoral neck, longitudinal trends in biochemical markers of bone turnover (CTX, ALP and PTH), subgroup analyses with particular focus on prior bisphosphonate exposure, and the occurrence of fractures during treatment.

### Sample size considerations

Because this was a retrospective observational study, sample size was not determined a priori. The analysis was based on all eligible patients receiving continuous denosumab for at least 10 years, and the primary endpoint was evaluated in those with an available total hip DXA assessment at year 10.

### Statistical analysis

Continuous variables were summarized as means and standard deviations, whereas categorical variables were reported as frequencies and percentages. Group comparisons were performed using Student’s *t*-test, Mann-Whitney *U* test, *χ*^2^ test, or Fisher’s exact test, as appropriate based on data distribution and sample size.

Percent changes in BMD at the three skeletal sites (lumbar spine, total hip, and femoral neck) were analyzed using linear mixed-effects models with random intercepts for subjects. Time was included as a categorical factor (0, 2, 4, 6, 8, and 10 years), and models were adjusted for baseline BMD (g/cm^2^), age, renal function (time-varying eGFR calculated using the CKD-EPI 2021 creatinine equation), presence of severe vertebral fractures at baseline, baseline prednisone-equivalent daily glucocorticoid dose, and time-varying prednisone-equivalent daily glucocorticoid dose during follow-up. Interaction terms between time and prior bisphosphonate exposure, as well as between time and incident fractures occurring during treatment, were introduced to explore potential effect modification. Fixed effects were evaluated using type III ANOVA with Satterthwaite approximation for degrees of freedom. Estimated marginal means (EMMs) of %BMD change, with 95% confidence intervals, were derived using the emmeans package. Pairwise contrasts between timepoints or between groups at each timepoint were computed with multiplicity-adjusted *p*-values using Bonferroni correction for all pairwise comparisons and Dunnett adjustment for comparisons versus baseline.

Longitudinal trends in bone turnover markers (CTX, ALP, and PTH) were analyzed using linear mixed-effects models with random intercepts, including time as a categorical factor and adjusting for eGFR as a time-varying covariate. Interaction terms between time and prior bisphosphonate exposure, and between time and incident fractures, were included to assess effect modification. Fixed effects were tested with type III ANOVA, and least-squares means with pairwise contrasts at each timepoint were computed using multiplicity-adjusted *p* values (Bonferroni correction) and Satterthwaite approximation for degrees of freedom.

All analyses were performed in R (version 4.4.3) using the lme4, lmerTest, and emmeans packages.

### Management of missing data

Missing values for DXA measurements and bone turnover markers were primarily attributable to the inherent variability of follow-up intervals in real-world clinical practice, patient availability for scheduled assessments, disruptions related to the SARS-CoV-2 pandemic, and the progressive reduction in observations at later treatment timepoints. As there was no evidence that the missingness was associated with densitometric or clinical outcomes, it was considered to follow a missing-at-random (MAR) mechanism. Analyses were therefore performed on the available data without imputation, leveraging the ability of mixed-effects models to accommodate unbalanced longitudinal datasets.

### Ethical approval

The study was conducted according to protocol 1483CESC approved by our local Ethics Committee (CET-ASOV, approval data: 07/10/2021), in accordance with the 1964 Helsinki Declaration and its later amendments or comparable ethical standards. Informed consent was collected for each participant.

## Results

### Study population

**Fig. 1:**
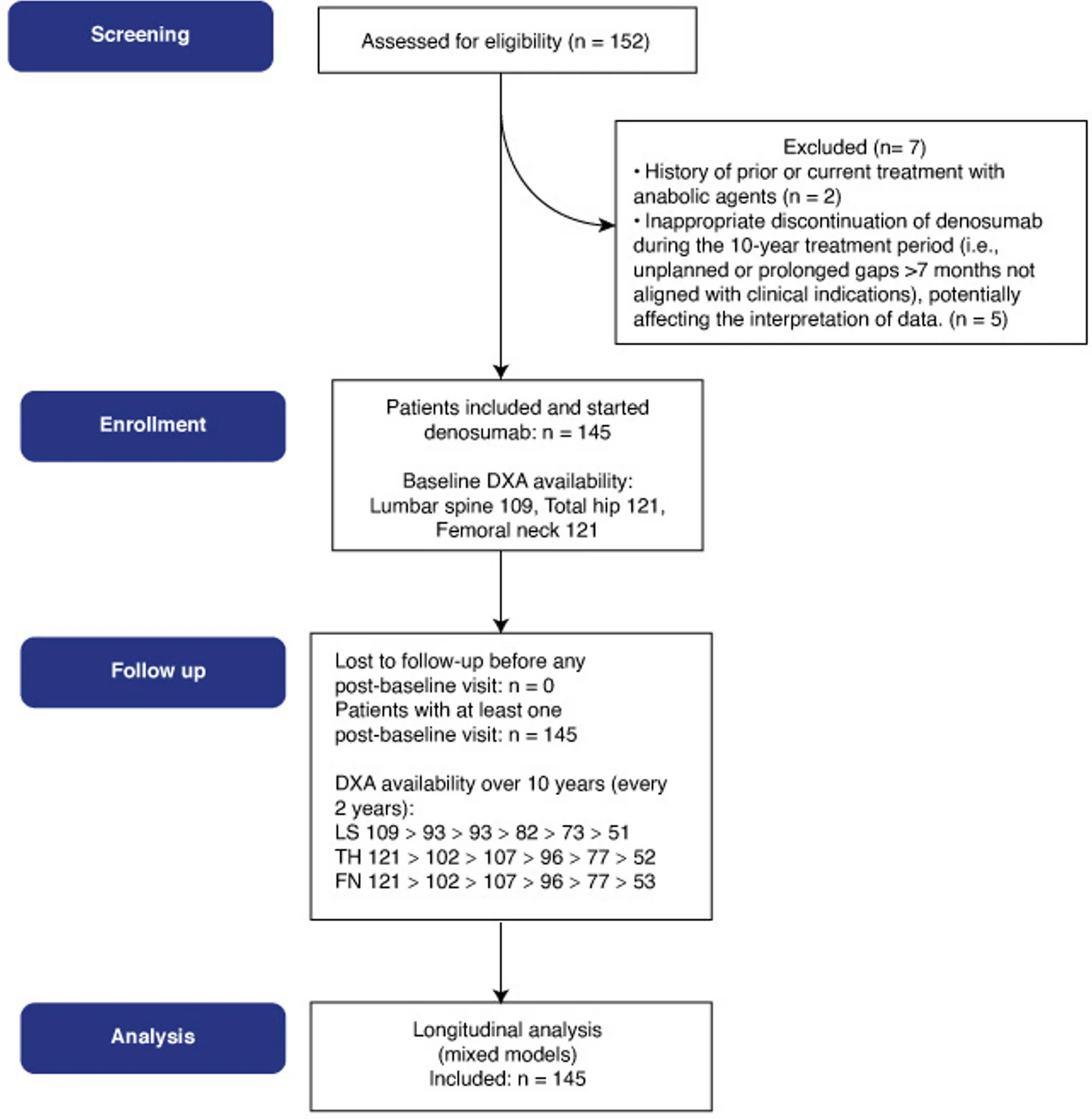
Study flow diagram

Data were collected from 145 patients who received at least 10 years of denosumab therapy between January 2012 and January 2026 (Fig. 1).

At baseline, DXA scans were available for 109 patients at the lumbar spine, 121 at the total hip, and 121 at the femoral neck. The number of assessments declined over time, with 51, 52, and 53 scans available at year 10, respectively. Across all timepoints, each patient contributed a mean of 4.0 ± 1.4 lumbar spine, 4.3 ± 1.3 total hip, and 4.2 ± 1.3 femoral neck DXA evaluations.

The cohort included 145 patients, predominantly women (95.9%), with a mean age of 70.9 years and BMI of 25.0 kg/m^2^. Mean denosumab exposure was 11.5 years. Prior bisphosphonate therapy was reported in 83 patients (57.2%), with a cumulative duration of 5.5 ± 4.3 years. Baseline demographic and clinical characteristics were comparable between bisphosphonate-naïve and previously treated patients, including renal function, smoking status, family history of osteoporosis, age at menopause, and major comorbidities. Chronic glucocorticoid use was present in approximately 20% of the cohort and did not differ significantly between groups (*p* = 0.134).

At baseline, serum 25OHD, ALP, and PTH levels were similar between groups. CTX levels were numerically higher in bisphosphonate-naïve patients (median [IQR], 0.39 [0.19–0.55] vs 0.20 [0.12–0.39] ng/mL), although this difference was not statistically significant (*p* = 0.176). The prevalence of vertebral fractures (*n* = 120, 82.8%) and femoral fractures (*n* = 22, 15.2%) did not differ between groups, and the mean number of vertebral fractures was similar.

Non-vertebral, non-femoral fractures were infrequent and similarly distributed between groups, including wrist fractures (6/62, 9.7% vs 18/83, 21.7%; *p* = 0.087), rib fractures (6/62, 9.7% vs 16/83, 19.3%; *p* = 0.174), and humerus fractures (3/62, 4.8% vs 3/83, 3.6%; *p* = 1.000). Baseline *T*-scores at the lumbar spine, total hip, and femoral neck were similar between groups, indicating a comparable densitometric profile at study entry. Full baseline characteristics are reported in Table 1.

**Table 1:** Demographic, clinical, laboratory, and densitometric characteristics of the study population. Continuous variables are reported as mean (SD) or median [IQR], as appropriate, and categorical variables as *n* (%). Comparisons between bisphosphonate-naïve and bisphosphonate-experienced patients were performed using the appropriate statistical tests; only *p*-values are reported in the table

	**All (*n* = 145)**	**Bisphosphonate naïve (*n* = 62)**	**Bisphosphonate experienced (*n* = 83)**	***p***
Age (years)	70.9 (7.6)	69.8 (8.3)	71.7 (6.9)	0.140
Female sex (*n*)	139 (95.9)	58 (93.5)	81 (97.6)	0.431
BMI (kg/m^2^)	25 (4.38)	25.33 (4.67)	24.72 (4.15)	0.465
Years on denosumab (years)	11.46 (1.46)	11.34 (1.45)	11.54 (1.47)	0.408
eGFR (mL/min)	82.49 (32.76)	87.99 (38.23)	78.25 (28.02)	0.364
Family history of osteoporosis (*n*)	44 (30.3)	19 (30.6)	25 (30.1)	1.000
Age at menopause (years)	47.20 (7.25)	48.18 (8.16)	46.53 (6.52)	0.217
Smoking status (*n*)	21 (14.6)	8 (12.9)	13 (15.9)	0.796
On chronic glucocorticoid therapy (*n*)	28 (19.3)	16 (25.8)	12 (14.5)	0.134
Prednisone-equivalent dose, mg/day, median [IQR]	2.50 [1.25–12.5]	2.50 [1.25–5]	2.50 [1.25–12.5]	0.119
Diabetes (*n*)	12 (8.3)	7 (11.3)	5 (6.0)	0.404
Rheumatoid arthritis (*n*)	13 (9.0)	7 (11.3)	6 (7.2)	0.580
25-OH vitamin D (ng/mL)	46.25 (19.46)	44.39 (23.16)	47.32 (17.29)	0.617
CTX (ng/mL), median [IQR]	0.219 [0.137–0.430]	0.389 [0.192–0.548]	0.200 [0.124–0.394] (0.21)	0.176
ALP (U/L), median [IQR]	64 [55.75–90.50]	65 [60–87]	63 [55–93]	0.886
PTH (pg/mL), median [IQR]	42.15 [25.25–52.32]	40.5 [31.1–45.7]	43.8 [22.6–56.6]	0.874
Presence of femoral fracture (*n*)	22 (15.2)	10 (16.1)	12 (14.5)	0.965
Presence of vertebral fracture (*n*)	120 (82.8)	51 (82.3)	69 (83.1)	1.000
Number of vertebral fracture (*n*)	1.97 (1.67)	1.89 (1.60)	2.04 (1.74)	0.598
Lumbar spine *T*-score	− 3.16 (0.94)	− 3.01 (0.93)	− 3.25 (0.94)	0.183
Femoral neck *T*-score	− 2.39 (0.81)	− 2.38 (0.73)	− 2.39 (0.86)	0.966
Total hip *T*-score	− 2.31 (1.46)	− 2.44 (2.12)	− 2.23 (0.85)	0.442

*BMI* body mass index, *eGFR* estimated glomerular filtration rate, *CTX* serum C-terminal telopeptide of type I collagen, *ALP* alkaline phosphatase, *PTH* parathyroid hormone, *25-OH vitamin D* 25-hydroxyvitamin D

### Ten-year change in bone mineral density

#### Primary outcome: total hip BMD

**Fig. 2:**
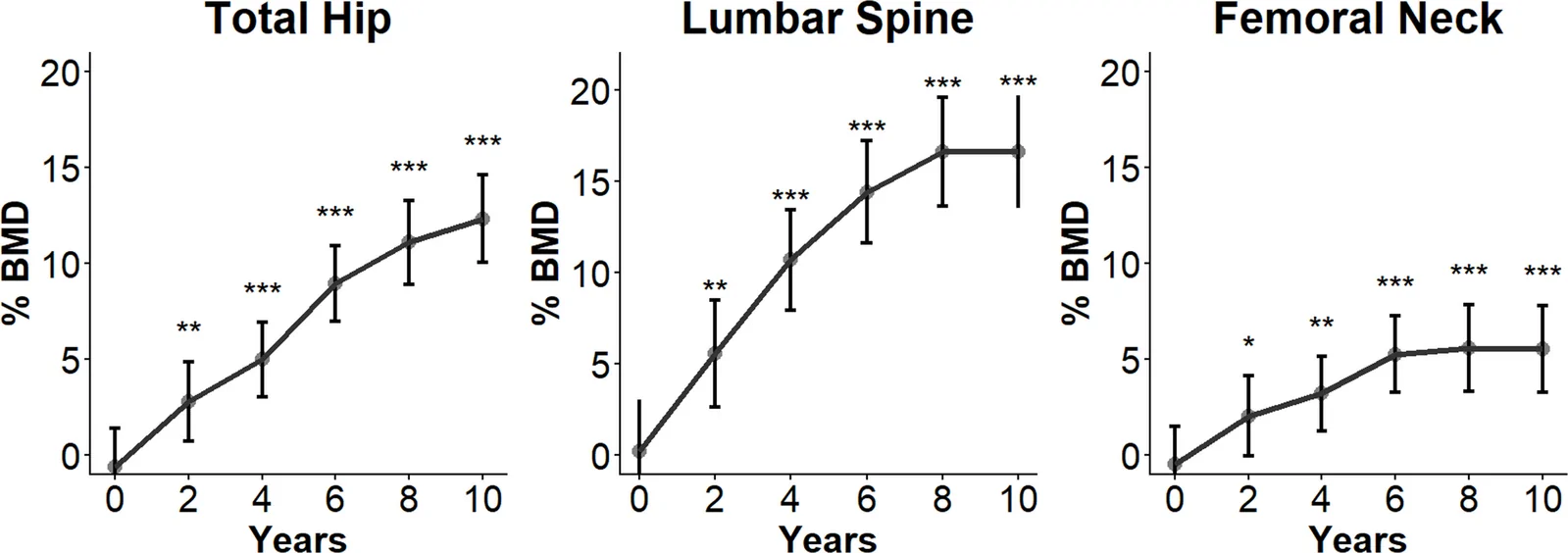
Percent change in BMD at the total hip, lumbar spine and femoral neck from baseline to 10 years. Points represent estimated marginal means derived from linear mixed-effects models; vertical bars indicate 95% confidence intervals. Asterisks indicate statistically significant differences versus baseline (*p < 0.05, **p < 0.01, ***p < 0.001) based on multiplicity-adjusted contrasts obtained from the mixed-effects models

Over the 10-year treatment period, total hip BMD showed a significant and sustained increase, with a strongly significant effect of time even after adjustment for baseline BMD, age, eGFR, severe vertebral fractures at baseline, and glucocorticoid exposure (Fig. 2). The trajectory was more gradual yet consistently upward, with increases of + 3.4% at year 2 (*p* < 0.009), + 5.6% at year 4 (*p* < 0.001), + 9.6% at year 6 (*p* < 0.001), + 11.7% at year 8 (*p* < 0.001), and + 12.9% at year 10 (*p* < 0.001).

#### Secondary outcomes: lumbar spine and femoral neck BMD

At the lumbar spine, BMD showed a marked and progressive increase as early as year 2 (+ 5.3%, *p* < 0.001), which continued at years 4 (+ 10.5%, *p* < 0.001), 6 (+ 14.2%, *p* < 0.001), and 8 (+ 16.4%, *p* < 0.001), reaching + 16.4% at year 10 (*p* < 0.001). At the femoral neck, gains were also significant, with changes of + 2.5% at year 2 (*p* = 0.05), + 3.7% at year 4 (*p* = 0.002), + 5.7% at year 6 (*p* < 0.001), + 6.1% at year 8 (*p* < 0.001), and + 6.0% at year 10 (*p* < 0.001).

In all models, baseline BMD was negatively associated with subsequent increase (lumbar spine: *β* = −21.68, *p* = 0.009; total hip: *β* = −25.66, *p* < 0.001; femoral neck: *β* = −39.48, *p* < 0.001), whereas age, eGFR, severe vertebral fractures at baseline, baseline glucocorticoid dose, and time-varying glucocorticoid exposure were not significantly associated with longitudinal BMD changes (all *p* > 0.240).

### Longitudinal trends in bone turnover markers

For PTH, the mixed-effects model did not show clinically or statistically significant changes relative to baseline. Estimated differences at individual timepoints were small and non-significant: + 4.8 pg/mL at year 2 (*p* = 0.638), −3.0 pg/mL at year 4 (*p* = 0.863), + 6.8 pg/mL at year 6 (*p* = 0.219), + 1.7 pg/mL at year 8 (*p* = 0.955), and −1.6 pg/mL at year 10 (*p* = 0.968).

CTX showed a marked reduction early during treatment, already significant at year 2 (−0.252 ng/mL; *p* < 0.001), and remained stable thereafter: −0.237 ng/mL at year 4, −0.185 ng/mL at year 6, −0.205 ng/mL at year 8, and −0.202 ng/mL at year 10 (all *p* < 0.001).

**Fig. 3:**
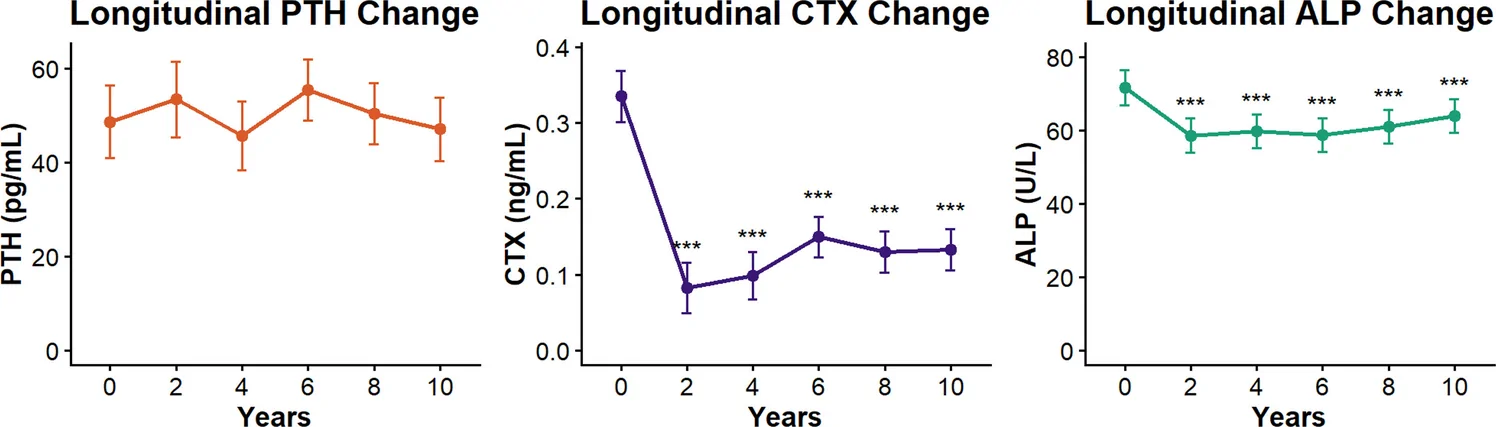
Longitudinal trends of bone turnover markers during 10 years of denosumab treatment. Mean changes over time in PTH (pmol/L), CTX (ng/mL), and ALP (U/L) are shown at baseline and at 2, 4, 6, 8, and 10 years. Error bars represent 95% confidence intervals (95% CI). Asterisks indicate statistically significant differences versus baseline (*p < 0.05, **p < 0.01, ***p < 0.001) based on multiplicity-adjusted contrasts obtained from the mixed-effects models

Total ALP also decreased significantly over time, with reductions evident at year 2 (−13.1 U/L; *p* < 0.001) and maintained at years 4 (−11.9 U/L), 6 (−12.9 U/L), 8 (−10.7 U/L), and at year 10 (− 7.7 U/L; all *p* < 0.001). Longitudinal trends for all bone turnover markers are shown in Fig. 3.

### Changes in BMD and bone turnover markers according to prior bisphosphonate exposure

**Fig. 4:**
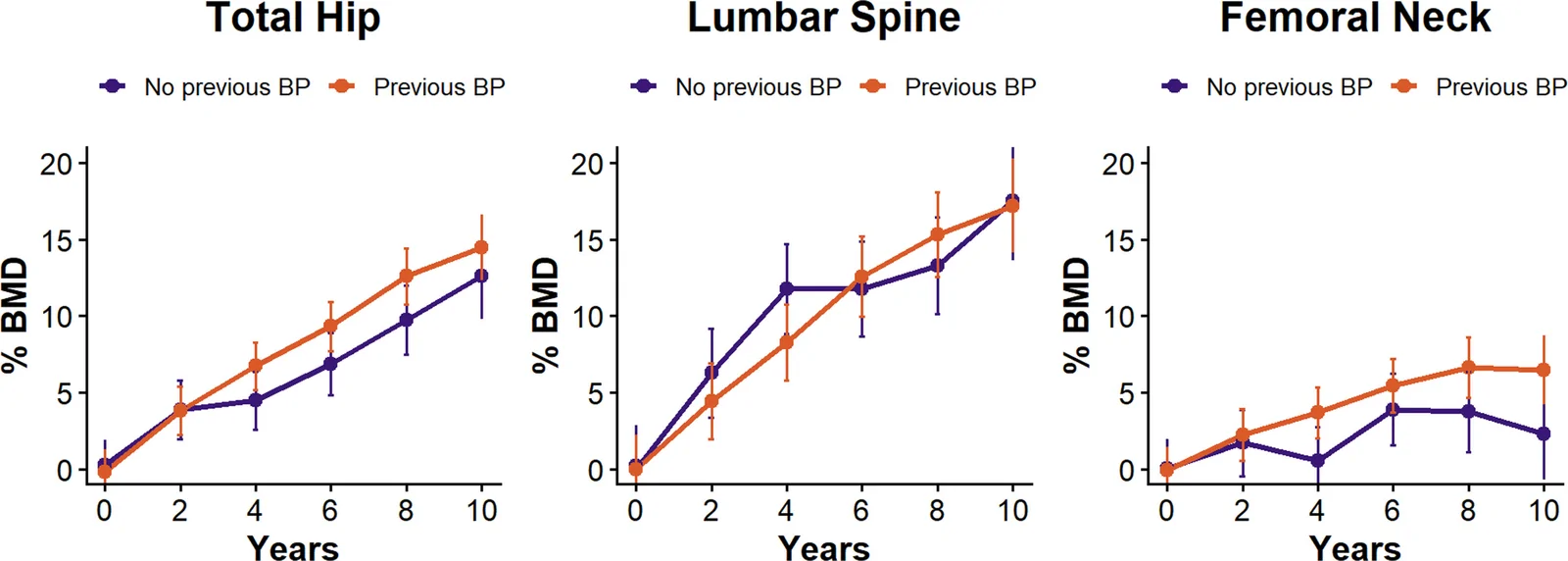
Percent change in BMD over time according to prior bisphosphonate exposure. Curves show estimated marginal means ± 95% CI for the total hip (left), lumbar spine (center), and femoral neck (right), derived from linear mixed-effects models. Asterisks indicate statistically significant differences between groups at individual timepoints based on multiplicity-adjusted contrasts from the mixed-effects models (*p < 0.05, **p < 0.01, ***p < 0.001)

Mixed-effects models showed no evidence that prior bisphosphonate exposure modified longitudinal BMD trajectories (Fig. 4). At the lumbar spine, the time × prior-exposure interaction was not significant (*p* = 0.189), with no main effect of prior therapy (*β* =  − 0.23%, *p* = 0.682).

At the total hip, results were similar, with a non-significant interaction (*p* = 0.148) and no significant main effect of prior exposure (*β* =  − 0.40%, *p* = 0.069).

At the femoral neck, neither the interaction (*p* = 0.217) nor the effect of time (*p* = 0.264) was significant. A small but significant main effect of prior therapy was observed (*β* =  − 0.18%, *p* = 0.019), indicating a modest time-independent difference between groups.

**Fig. 5:**
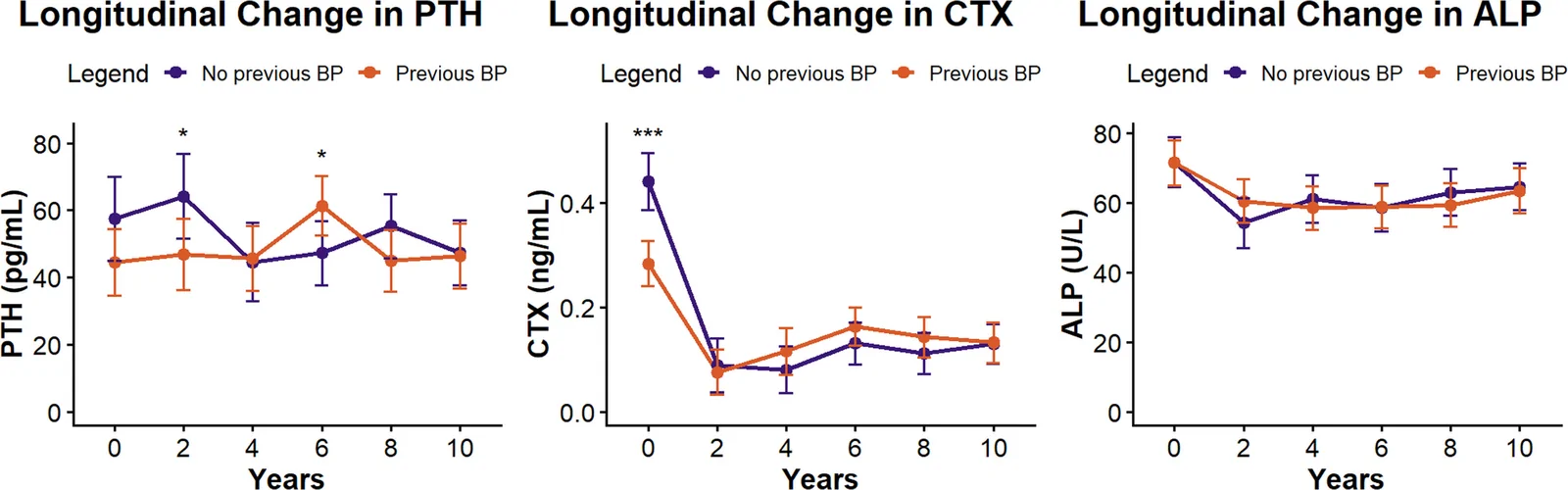
Longitudinal trends of bone turnover markers stratified by prior bisphosphonate exposure. Curves represent estimated marginal means ± 95% CI of PTH (left), CTX (center), and ALP (right) at 0, 2, 4, 6, 8, and 10 years of denosumab treatment, derived from linear mixed-effects models. Asterisks indicate statistically significant differences between groups at individual timepoints based on multiplicity-adjusted contrasts from the mixed-effects models (*p < 0.05, **p < 0.01, ***p < 0.001)

Prior bisphosphonate exposure had heterogeneous effects on longitudinal bone turnover marker trajectories (Fig. 5).

For PTH, the time × prior-exposure interaction was significant (*p* < 0.001), but differences at individual timepoints were small and mostly nonsignificant (baseline *p* = 0.110; year 4 *p* = 0.897; year 8 *p* = 0.130; year 10 p = 0.880), with significant contrasts only at year 2 (+ 17.3 pg/mL; *p* = 0.040) and year 6 (− 14.1 pg/mL; *p* = 0.035).

For CTX, the interaction was also significant (*p* < 0.001). Model-based estimates indicated lower baseline levels in patients previously treated with bisphosphonates (− 0.157 ng/mL; *p* < 0.001). Trajectories progressively converged, with comparable reduction during follow-up. Changes from baseline were significant at all timepoints (all *p* < 0.001), with no between-group differences thereafter (all *p* > 0.48).

For ALP, the interaction was significant (*p* = 0.008), without a main effect of prior exposure (*p* = 0.97). No relevant differences were observed at individual timepoints, except for a modest difference at year 2 (+ 6.5 U/L; *p* = 0.034).

### Changes in BMD and bone turnover in patients who sustained incident fractures during denosumab treatment

In the entire cohort, 15 fracture events were recorded, occurring at a median of 8 years (IQR 7–10). Most events were vertebral fractures (9/15, 60%). Non-vertebral fractures were less frequent and occurred at different skeletal sites, including two hip fractures, two femoral fractures (one of which was atypical and occurred in a patient without prior bisphosphonate exposure), one wrist fracture, and one rib fracture.

**Fig. 6:**
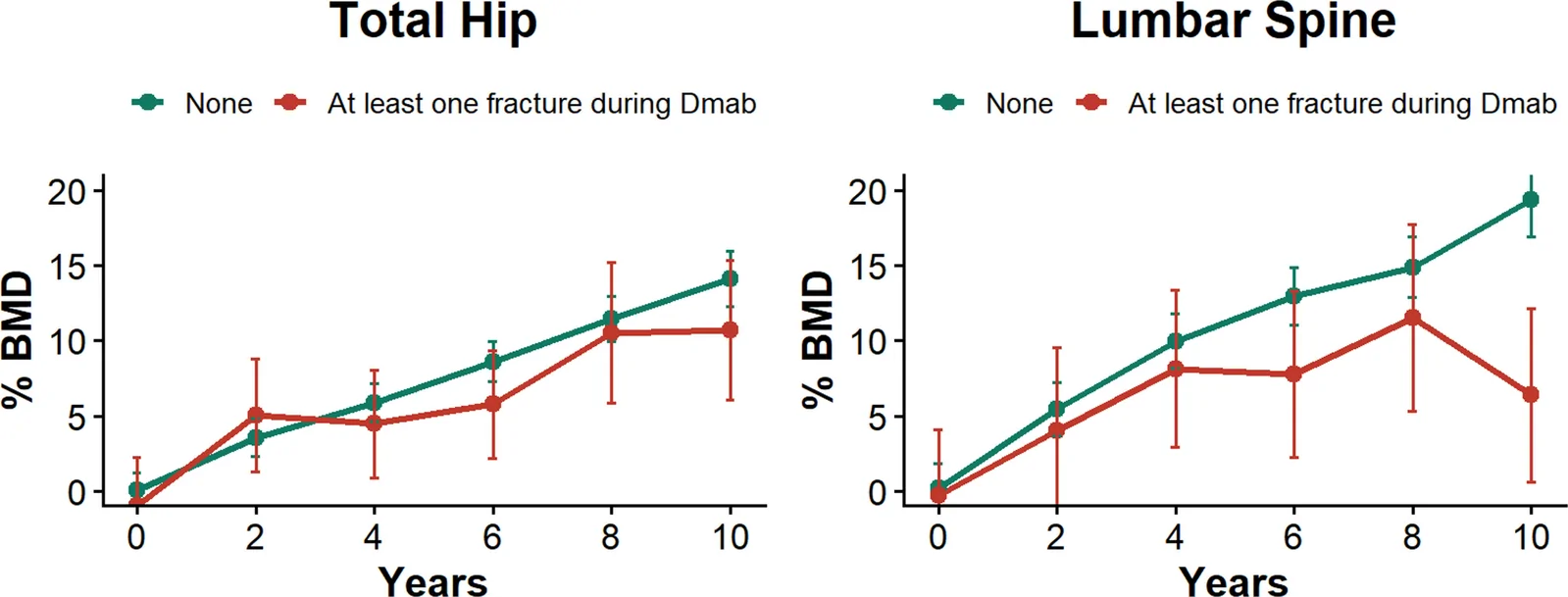
Percent change in BMD in patients with and without incident fractures during denosumab treatment. Curves represent estimated marginal means ± 95% CI for the total hip (left) and lumbar spine (right), derived from linear mixed-effects models. Asterisks indicate statistically significant differences between groups at individual timepoints based on multiplicity-adjusted contrasts from the mixed-effects models (*p < 0.05, **p < 0.01, ***p < 0.001)

Baseline clinical and densitometric characteristics were comparable between patients who experienced fractures during treatment and those who did not, with no significant differences between groups. Longitudinal BMD trajectories differed by skeletal site (Fig. 6).

At the lumbar spine, both the main effect of incident fracture (*p* = 0.022) and the time × fracture interaction (*p* = 0.010) were significant, with the largest difference at year 10 (interaction *β* =  − 12.56%, *p* < 0.001). At the total hip, neither the main effect (*p* = 0.291) nor the interaction (*p* = 0.499) was significant. Similarly, no significant effects were observed at the femoral neck (main effect *p* = 0.269; interaction *p* = 0.148). Overall, incident fractures were associated with reduced BMD gains at the lumbar spine only.

For bone turnover markers, CTX trajectories differed between patients with and without fractures. The time × fracture interaction was significant (*p* < 0.001). Patients who fractured had lower baseline CTX levels (− 0.201 ng/mL; *p* < 0.001), whereas no significant differences were observed at later timepoints, except for a difference at year 8 (− 0.074 ng/mL; *p* = 0.051).

## Discussion

In this real-world cohort of patients who remained on denosumab therapy for at least 10 years, we observed sustained and clinically meaningful increases in BMD at all skeletal sites, together with a low incidence of fractures during follow-up. These findings support the long-term effectiveness of continued denosumab therapy in patients who maintain treatment in routine clinical practice.

The magnitude and pattern of BMD gains were broadly consistent with long-term findings from the FREEDOM program and its extension [7, 8], despite the substantial differences between trial populations and our heterogeneous real-world cohort. In FREEDOM, cumulative 10-year increases reached + 21.7% at the lumbar spine, + 9.2% at the hip, and + 9.0 at the femoral neck. Our results (+ 17.3% at the spine, + 13.8% at the total hip, and + 4.9% at the femoral neck) fall within the expected range. It should be noted, however, that our cohort was older, more clinically heterogeneous, and included both men and patients with prior bisphosphonate exposure, features that were not represented in the original registration trial. These differences likely contributed to the greater variability observed while simultaneously improving the real-world generalizability of our findings. Moreover, total hip and femoral neck BMD should not be considered interchangeable in longitudinal analyses. In our cohort, both sites were systematically collected and analyzed; however, differences in the magnitude of change may partly reflect inherent site-specific and technical variability of DXA measurements, including positioning and region-of-interest factors, as described in the literature on operator-related pitfalls [13].

In one of the few available real-world cohorts with longitudinal evaluation after ≥ 20 doses [14], continued treatment was associated with further lumbar spine BMD gains and preservation of femoral BMD, whereas switching to zoledronic acid led to partial loss of prior improvements. These observations are in agreement with the sustained densitometric response observed in our cohort and support the concept that prolonged denosumab therapy may maintain skeletal benefits over time in selected high-risk patients.

Renal function has been suggested as a potential determinant of densitometric response to denosumab in some real-world cohorts [15]. However, post hoc analyses of randomized trials and studies in patients with chronic kidney disease [16, 17] have shown that BMD gains are generally preserved across different levels of renal function. In line with these observations, we did not find an independent association between time-varying eGFR and longitudinal BMD changes in mixed-effects models.

Biochemical data also aligned with previous evidence. CTX showed marked and durable reduction over the entire follow-up, with minor oscillations in later years similar to those described in randomized clinical trial [8, 18]. ALP reductions stabilized over time, without signs of suppression of bone formation. PTH levels did not show significant long-term changes, a pattern consistent with the transient parathyroid response known to occur after each denosumab injection [19–21]. PTH typically rises only in the weeks following administration and returns to baseline before the next 6-month dose [22]. In a real-world setting with biannual sampling and with patients with normal renal function, as in our study, measurements are more likely to capture this post-peak steady-state phase rather than the initial transient increase, thus explaining the absence of detectable longitudinal differences.

Prior bisphosphonate exposure is a well-known factor influencing early densitometric response to anti-osteoporotic therapy [23–26]. Several prospective cohorts have shown that pre-treatment with bisphosphonates is associated with smaller BMD gains during the first 12–24 months of denosumab [27–30]. This effect may reflect both persistent reduction of bone remodeling and the fact that part of the remodeling space may have already been filled during prior antiresorptive therapy, resulting in a reduced potential for further BMD increase after switching to denosumab [31, 32]. Other studies, however, did not detect significant differences between naïve and pre-treated patients in the first year of therapy [33]. Importantly, all these findings refer to the early response phase (12–24 months). Our long-term real-world data extend this evidence by demonstrating that prior bisphosphonate exposure does not modify the densitometric trajectory over 10 years. In our cohort, no significant differences were observed at the lumbar spine, total hip, or femoral neck, indicating comparable long-term BMD trajectories in previously untreated and previously treated patients. Overall, our results suggest that prior bisphosphonate exposure does not compromise the cumulative benefit of prolonged denosumab therapy.

Baseline differences in bone turnover, consistent with prior bisphosphonate exposure [29], progressively disappeared after treatment initiation, indicating that denosumab rapidly reduces bone resorption to a similar extent in both groups, with comparable CTX levels already from the first injections [34]. Importantly, previously treated patients did not show a greater degree of CTX reduction, suggesting that prior bisphosphonate exposure does not lead to excessive inhibition of bone turnover. In both groups, CTX values during treatment fell within the range expected during effective antiresorptive therapy and were consistent with the premenopausal reference values commonly used as treatment targets for postmenopausal osteoporosis [35].

Another interesting finding is that, among patients who sustained fractures during treatment, BMD trajectories showed some divergence from those of non-fractured patients, mainly at the lumbar spine. Only this site displayed a significantly attenuated BMD gain, whereas total hip and femoral neck BMD progressed similarly in both groups. Given that most fractures were vertebral, a site-specific difference at the lumbar spine is not unexpected. However, this observation should be interpreted with caution, as it is based on a limited number of events and was mainly evident at late timepoints, where the number of observations was reduced, making it difficult to establish a causal relationship between attenuated BMD response and fracture occurrence.

In any case, the number of fractured patients in our cohort was relatively small despite the long follow-up, even though access to denosumab in Italy during the study period was generally restricted to patients at high fracture risk according to reimbursement criteria. Previous real-world data have indeed shown that patients receiving denosumab in Italy often have a particularly severe fracture risk profile, which may influence both treatment selection and outcomes [36]. It is possible that patients who fractured had a higher baseline fragility risk, related to factors not captured by BMD alone, such as age, fall risk, comorbidities, or skeletal quality, which could also contribute to a less pronounced densitometric response.

Post hoc analyses of the FREEDOM program have shown that, overall, greater on-treatment increases in BMD and higher achieved total hip *T*-scores are associated with lower fracture rates under denosumab, supporting the use of BMD as a surrogate of fracture risk [37, 38]. In routine clinical practice, registry-based data have similarly shown that changes in BMD during therapy are associated with the magnitude of fracture risk reduction, supporting the concept that densitometric response reflects treatment effectiveness [39]. However, specific data on site-specific BMD trajectories in patients who fracture while on therapy are limited. In our cohort, fractures were associated with a numerically attenuated lumbar spine BMD gain, with no differences at hip sites; this pattern should be interpreted as exploratory rather than indicative of a predictive effect.

By design, the present study included only patients who maintained continuous denosumab therapy for at least 10 years. Therefore, the findings should be interpreted as describing the long-term skeletal trajectories of treatment completers rather than estimating treatment effectiveness in the overall treated population. While this design may introduce survivorship bias, it allowed us to specifically characterize the skeletal response achievable under sustained therapy in routine clinical practice.

### Limitations

This study has several limitations that should be considered when interpreting the results. Its retrospective design inherently introduces potential selection bias and limits the ability to infer causal relationships. In addition, we included only patients who completed at least 10 years of continuous denosumab therapy, which introduces an inherent selection of long-term treatment completers. This approach may introduce survivorship bias and may overestimate treatment-related BMD gains when extrapolated to the overall treated population. Notably, in a minority of cases in which fractures occurred during treatment, continuation of denosumab reflected real-world clinical decision-making, often influenced by patient preference or refusal of alternative therapies, rather than a predefined protocol. In addition, the relatively small number of fracture events and subgroup sample sizes limits the precision of these analyses; therefore, these findings should be interpreted as exploratory and hypothesis-generating. Our aim, however, was not to estimate treatment effectiveness in the overall treated population, but rather to describe the long-term clinical trajectory of patients who maintain denosumab therapy for a decade in routine practice.

Although the real-world nature of the study is a strength in terms of generalizability, it also implies reduced control over treatment adherence and biochemical monitoring. This may have contributed to variability in the observed measurements. External factors, such as the COVID-19 pandemic, likely impacted follow-up attendance for logistical reasons and contributed to missing data around 2020.

Finally, the absence of a control group, either untreated patients or individuals receiving alternative osteoporosis therapies, limits the ability to directly compare outcomes and quantify the relative effect of long-term denosumab treatment.

## Conclusions

In this real-world cohort of patients receiving continuous denosumab for 10 years, treatment was associated with sustained and clinically meaningful increases in BMD at all skeletal sites together with durable reduction of bone turnover. Prior bisphosphonate exposure did not modify long-term densitometric trajectories. Overall, these findings support the long-term effect of continued denosumab in routine clinical practice and provide additional insight into factors associated with heterogeneous skeletal responses during prolonged therapy.

## Data Availability

Data of the analysis is available upon reasonable request.

## References

[1] Corrao G, Biffi A, Porcu G, Ronco R, Adami G, Alvaro R et al (2021) Executive summary: Italian guidelines for diagnosis, risk stratification, and care continuity of fragility fractures 2021. Front Endocrinol 14:1137671. 10.3389/fendo.2023.1137671 10.3389/fendo.2023.1137671 PMC10151776 37143730

[2] Adami G, Tsourdi E, Rossini M, Funck-Brentano T, Chapurlat R (2023) Patients with osteoporosis: children of a lesser god. RMD Open 9:e002973. 10.1136/rmdopen-2022-002973 36759006 10.1136/rmdopen-2022-002973 PMC9923352

[3] Lamy O, Everts-Graber J, Rodriguez EG (2025) Denosumab for osteoporosis treatment: when, how, for whom, and for how long. A pragmatical approach. Aging Clin Exp Res 37:70. 10.1007/s40520-025-02991-z 40055268 10.1007/s40520-025-02991-z PMC11889064

[4] Fassio A, Gatti D, Bertelle D, Fracassi E, Zanetti G, Viapiana O et al (2022) Comparable long-term retention rates and effects on bone mineral density of denosumab treatment in patients with osteoporosis with or without autoimmune inflammatory rheumatic diseases: real-life data. Ther Adv Musculoskelet Dis 14:1759720X221124543. 10.1177/1759720X221124543 36158710 10.1177/1759720X221124543 PMC9490481

[5] Adami G, Gavioli I, Rossini M, Viapiana O, Orsolini G, Benini C et al (2022) Real-life short-term effectiveness of anti-osteoporotic treatments: a longitudinal cohort study. Ther Adv Musculoskelet Dis 14:1759720X221105009. 10.1177/1759720X221105009 35784611 10.1177/1759720X221105009 PMC9243369

[6] Adami G, Fassio A, Gatti D, Viapiana O, Benini C, Danila MI, et al (2022) Osteoporosis in 10 years time: a glimpse into the future of osteoporosis. Therapeutic Advances in Musculoskeletal ;14:1759720X221083541. 10.1177/1759720X221083541. 10.1177/1759720X221083541 PMC8941690 35342458

[7] Papapoulos S, Lippuner K, Roux C, Lin CJF, Kendler DL, Lewiecki EM et al (2015) The effect of 8 or 5 years of denosumab treatment in postmenopausal women with osteoporosis: results from the FREEDOM Extension study. Osteoporos Int 26:2773–83. 10.1007/s00198-015-3234-7 26202488 10.1007/s00198-015-3234-7 PMC4656716

[8] Bone HG, Wagman RB, Brandi ML, Brown JP, Chapurlat R, Cummings SR et al (2017) 10 years of denosumab treatment in postmenopausal women with osteoporosis: results from the phase 3 randomised FREEDOM trial and open-label extension. Lancet Diabetes Endocrinol 5:513–523. 10.1016/S2213-8587(17)30138-9 28546097 10.1016/S2213-8587(17)30138-9

[9] Cummings SR, Martin JS, McClung MR, Siris ES, Eastell R, Reid IR et al (2009) Denosumab for prevention of fractures in postmenopausal women with osteoporosis. N Engl J Med 361:756–765. 10.1056/NEJMoa0809493 19671655 10.1056/NEJMoa0809493

[10] Kendler DL, Cosman F, Stad RK, Ferrari S (2022) Denosumab in the treatment of osteoporosis: 10 years later: a narrative review. Adv Ther 39:58–74. 10.1007/s12325-021-01936-y 34762286 10.1007/s12325-021-01936-y PMC8799550

[11] Krueger D, Tanner SB, Szalat A, Malabanan A, Prout T, Lau A et al (2023) DXA reporting updates: 2023 Official Positions of the International Society for Clinical Densitometry. J Clin Densitom 27:101437. 10.1016/j.jocd.2023.101437 38011777 10.1016/j.jocd.2023.101437

[12] Vilaca T, Lui L-Y, Schini M, Ewing SK, Thompson A, Vittinghoff E et al (2025) Treatment-related changes in total hip bone mineral density are applicable to trials of varied study designs and to drugs with differing mechanisms of action: meta-regression results from the FNIH-ASBMR SABRE study. J Bone Miner Res 40:1228–37. 10.1093/jbmr/zjaf100 40720735 10.1093/jbmr/zjaf100 PMC12578277

[13] Albano D, Agnollitto PM, Petrini M, Biacca A, Ulivieri FM, Sconfienza LM et al (2021) Operator-related errors and pitfalls in dual energy X-ray absorptiometry: how to recognize and avoid them. Acad Radiol 28:1272–86. 10.1016/j.acra.2020.07.028 32839098 10.1016/j.acra.2020.07.028

[14] Xiong X, Wong CH, Tsoi KH, Loong CHN, Fong CHY, Lee ACH et al (2026) Denosumab therapy beyond 10 years: subsequent treatment and densitometric outcomes. Endocr Pract. 10.1016/j.eprac.2026.01.751 41643813 10.1016/j.eprac.2026.01.751

[15] Catalano A, Oliveri C, Natale G, Agostino RM, Squadrito G, Gaudio A et al (2024) Renal function is associated with changes in bone mineral density in postmenopausal osteoporotic women treated with denosumab: data from a retrospective cohort study. J Clin Med 13:6239. 10.3390/jcm13206239 39458189 10.3390/jcm13206239 PMC11514604

[16] Broadwell A, Chines A, Ebeling PR, Franek E, Huang S, Smith S et al (2021) Denosumab safety and efficacy among participants in the FREEDOM extension study with mild to moderate chronic kidney disease. J Clin Endocrinol Metab 106:397–409. 10.1210/clinem/dgaa851 33211870 10.1210/clinem/dgaa851 PMC7823314

[17] Jamal SA, Ljunggren Ö, Stehman-Breen C, Cummings SR, McClung MR, Goemaere S et al (2011) Effects of denosumab on fracture and bone mineral density by level of kidney function. J Bone Miner Res 26:1829–1835. 10.1002/jbmr.403 21491487 10.1002/jbmr.403

[18] McClung MR, Lewiecki EM, Geller ML, Bolognese MA, Peacock M, Weinstein RL et al (2013) Effect of denosumab on bone mineral density and biochemical markers of bone turnover: 8-year results of a phase 2 clinical trial. Osteoporos Int 24:227–235. 10.1007/s00198-012-2052-4 22776860 10.1007/s00198-012-2052-4 PMC3536967

[19] Rotman-Pikielny P, Barzilai-Yosef L, Ramaty E, Braginski-Shapira S, Meron MK, Lurie TH (2025) Parathyroid hormone levels following denosumab vs. zoledronic acid therapy for osteoporosis. Bone 193:117407. 10.1016/j.bone.2025.117407 39863008 10.1016/j.bone.2025.117407

[20] Lee JH, Heo S-J, Kim KM, Lee JE (2025) Trends in serum phosphate, calcium, and parathyroid hormone levels according to renal function following denosumab. Osteoporos Int 36:1661–1669. 10.1007/s00198-025-07601-2 40650741 10.1007/s00198-025-07601-2

[21] Fassio A, Andreola S, Gatti D, Pollastri F, Gatti M, Fabbrini P et al (2024) Long-term bone mineral density changes in kidney transplant recipients treated with denosumab: a retrospective study with nonequivalent control group. Calcif Tissue Int 115:23–30. 10.1007/s00223-024-01218-z 38730099 10.1007/s00223-024-01218-z PMC11153264

[22] Gatti D, Viapiana O, Fracassi E, Idolazzi L, Dartizio C, Povino MR et al (2012) Sclerostin and DKK1 in postmenopausal osteoporosis treated with denosumab. J Bone Miner Res 27:2259–63. 10.1002/jbmr.1681 22692843 10.1002/jbmr.1681

[23] Cosman F, Langdahl B, Leder BZ (2024) Treatment sequence for osteoporosis. Endocr Pract 30:490–496. 10.1016/j.eprac.2024.01.014 38311211 10.1016/j.eprac.2024.01.014

[24] Nayak S, Greenspan SL (2026) A systematic review and meta-analysis of sequential treatment strategies for osteoporosis. Osteoporos Int 37:1–13. 10.1007/s00198-025-07717-5 41105226 10.1007/s00198-025-07717-5

[25] Fassio A, Gatti D, Biffi A, Ronco R, Porcu G, Adami G et al (2024) The sequential antifracturative treatment: a meta-analysis of randomized clinical trials. Ther Adv Musculoskelet Dis 16:1759720X241234584. 10.1177/1759720X241234584 38654732 10.1177/1759720X241234584 PMC11036926

[26] Tsourdi E, Zillikens MC, Meier C, Body J-J, Gonzalez Rodriguez E, Anastasilakis AD et al (2021) Fracture risk and management of discontinuation of denosumab therapy: a systematic review and position statement by ECTS. J Clin Endocrinol Metab 106:264–81. 10.1210/clinem/dgaa756 10.1210/clinem/dgaa756 33103722

[27] Kendler DL, Roux C, Benhamou CL, Brown JP, Lillestol M, Siddhanti S et al (2010) Effects of denosumab on bone mineral density and bone turnover in postmenopausal women transitioning from alendronate therapy. J Bone Miner Res 25:72–81. 10.1359/jbmr.090716 19594293 10.1359/jbmr.090716

[28] Jiang X, Hou S, Deng X, Hu L, Wang J, Hou D (2024) Sequential treatment from bisphosphonate to denosumab improves lumbar spine bone mineral density in postmenopausal osteoporosis patients: a meta-analysis of randomized controlled trials. Medicine (Baltimore) 103:e40594. 10.1097/MD.0000000000040594 39560527 10.1097/MD.0000000000040594 PMC11576034

[29] Guan G, Du Y, Tang W, Chen M, Yu W, Li H et al (2025) Impacts of prior anti-osteoporosis treatments on sequential denosumab responses in BMD changes among postmenopausal osteoporosis women in East China: real-world data analysis. CIA 20:573–586. 10.2147/CIA.S511622 10.2147/CIA.S511622 PMC12068388 40357344

[30] Yamamoto A, Nagao M, Nishizaki Y, Maeda E, Ishijima M (2024) Risk factors for nonresponse to 2 years of denosumab administration in patients with osteoporosis: a retrospective single-center cohorts study. Health Sci Rep 7:e1993. 10.1002/hsr2.1993 38585014 10.1002/hsr2.1993 PMC10995440

[31] Ma YL, Zeng QQ, Chiang AY, Burr D, Li J, Dobnig H et al (2014) Effects of teriparatide on cortical histomorphometric variables in postmenopausal women with or without prior alendronate treatment. Bone 59:139–147. 10.1016/j.bone.2013.11.011 24269280 10.1016/j.bone.2013.11.011

[32] Bustamante-Gomez C, Fu Q, Goellner JJ, Thostenson JD, Reyes-Pardo H, O’Brien CA (2025) Potent suppression of bone remodeling by denosumab does not blunt the anabolic response to romosozumab in mice. Bone 199:117567. 10.1016/j.bone.2025.117567 40532840 10.1016/j.bone.2025.117567 PMC12236991

[33] Sánchez A, Brun LR, Salerni H, Costanzo PR, González D, Bagur A et al (2016) Effect of denosumab on bone mineral density and markers of bone turnover among postmenopausal women with osteoporosis. J Osteoporos 2016:1–6. 10.1155/2016/8738959 10.1155/2016/8738959 PMC4992763 27579211

[34] Anastasilakis AD, Polyzos SA, Efstathiadou ZA, Savvidis M, Sakellariou GT, Papatheodorou A et al (2015) Denosumab in treatment-naïve and pre-treated with zoledronic acid postmenopausal women with low bone mass: effect on bone mineral density and bone turnover markers. Metabolism 64:1291–1297. 10.1016/j.metabol.2015.06.018 26198440 10.1016/j.metabol.2015.06.018

[35] Adami S, Bianchi G, Brandi ML, Giannini S, Ortolani S, DiMunno O et al (2008) Determinants of bone turnover markers in healthy premenopausal women. Calcif Tissue Int 82:341–7. 10.1007/s00223-008-9126-5 18470550 10.1007/s00223-008-9126-5

[36] Adami G, Giollo A, Rossini M, Orsolini G, Benini C, Viapiana O et al (2020) Different fracture risk profile in patients treated with anti-osteoporotic drugs in real-life. Reumatismo 72:71–4. 10.4081/reumatismo.2020.1267 32700872 10.4081/reumatismo.2020.1267

[37] Austin M, Yang Y-C, Vittinghoff E, Adami S, Boonen S, Bauer DC et al (2012) Relationship between bone mineral density changes with denosumab treatment and risk reduction for vertebral and nonvertebral fractures. J Bone Miner Res 27:687–693. 10.1002/jbmr.1472 22095631 10.1002/jbmr.1472 PMC3415619

[38] Kendler DL, Chines A, Brandi ML, Papapoulos S, Lewiecki EM, Reginster J-Y et al (2019) The risk of subsequent osteoporotic fractures is decreased in subjects experiencing fracture while on denosumab: results from the FREEDOM and FREEDOM extension studies. Osteoporos Int 30:71–78. 10.1007/s00198-018-4687-2 30244369 10.1007/s00198-018-4687-2 PMC6331737

[39] Leslie WD, Majumdar SR, Morin SN, Lix LM (2016) Change in bone mineral density is an indicator of treatment-related antifracture effect in routine clinical practice: a registry-based cohort study. Ann Intern Med 165:465–472. 10.7326/M15-2937 27428723 10.7326/M15-2937

